# Brief report: Parental attitudes and knowledge on routine childhood immunization: an experience from Central Sri Lanka

**DOI:** 10.1186/s13104-018-3519-y

**Published:** 2018-06-22

**Authors:** N. C. Herath, T. Kudagammana, T. T. Sanathchandra, H. K. Gamage, I. M. Razik, V. Liynapathirana

**Affiliations:** 1 Department of Radiography, Faculty of Allied Health Sciences, University of Peradeniya, Peradeniya, Sri Lanka; 2 Department of Paediatrics, Faculty of Medicine, University of Peradeniya, Peradeniya, Sri Lanka; 3 Faculty of Medicine, University of Peradeniya, Peradeniya, Sri Lanka; 4 Department of Microbiology, Faculty of Medicine, University of Peradeniya, Peradeniya, Sri Lanka

**Keywords:** Vaccination, Immunization, Sri Lanka, Awareness, Hesitancy

## Abstract

**Objectives:**

A lack of correct awareness about immunization among parents put them at risk of falling prey to the anti-vaccine movement. This risk is present even in countries with a high vaccine uptake. This study was done with the objective of assessing the awareness of parents childhood vaccination.

**Results:**

In this study conducted among 141 parents accompanying children to a routine clinic we found that 53.2% of the participants had average or above average knowledge. Level of knowledge was associated with the level of education (OR: 2.7, 95% CI 1.4–5.4) and the sex of the parent (OR: 3.4, 95% CI 1.2–9.3). While our sample size is small, we recommend educational programmes for parents to strengthen their knowledge on vaccination to safeguard the continuity of a successful control of vaccine preventable diseases.

## Introduction

There is a global call to achieve more than 90% coverage of all vaccines available through the national immunization programmes of individual countries by 2020 [1]. However, there is an increasing concern about vaccine hesitancy defined as “delay in acceptance or refusal of vaccines despite availability of vaccinations services” [2]. Vaccine refusal or hesitancy is likely to contribute to major public health as well as economic consequences [3]. Sri Lanka has a very high vaccine coverage approaching 99% for most infectious diseases according to the WHO vaccine-preventable diseases [4].

Anecdotal stories of vaccine refusal are being reported from Sri Lanka. Lack of scientific knowledge regarding vaccination makes parents of young children vulnerable for misinformation and disbeliefs that may promote them to refuse vaccination for their children [5].

## Main text

This study was conducted with the objective of identifying the level of understanding among parents about childhood vaccination. Ethical Clearance was obtained from the Institutional Ethics Review Committee of the Faculty of Medicine, University of Peradeniya and informed written consent was taken from the participants.

A pre-validated interviewer administered questionnaire was used to assess the knowledge, attitudes, practices and concerns among 141 parents presenting with children to the well-baby clinic at Teaching Hospital, Peradeniya from September to November 2017. Data thus collected were analyzed using descriptive statistics, t and *X*^2^ tests with Odds ratios as applicable.

Majority (120, 85.1%) of the participants were mothers accompanying babies and the mean age of all participants was 28.9 years (SD 5.5 years) (Table 1).

**Table 1:** Demographic characteristics of the study participants

Characteristic	N (%) or mean (SD)
Age	28.9 years (5.5)
Gender	
Females	120 (85.1%)
Males	21 (14.9%)
Ethnicity	
Sinhalese	104 (73.8%)
Moor	30 (21.3%)
Tamil	7 (5%)
Maximum educational status achieved by the parent	
Not schooled	0
Primary	6 (4.3%)
O/L	61 (43.3%)
A/L	55 (39.0%)
Graduate	17 (12.1%)
Postgraduate	2 (1.4%)
Employment status of participating parents	
Employed	100 (70.9%)
Unemployed	41 (29.1%)
No of children	
1	58 (41.1%)
2	49 (34.8%)
3	28 (19.9%)
4 and above	6 (4.2%)
Monthly income of the family	
< 25,000 Rs	30 (21.3%)
25,000–50,000 Rs	71 (50.4%)
> 50,000 Rs	36 (25.5%)
Data not revealed	4 (2.8%)

Majority of the participants (127, 90.1%) thought that vaccinating their children is very important. The major motivation for parents to vaccinate children was their understanding that vaccination prevents serious disability (139, 98.6%). Other factors that contributed as factors promoting parents to take children for vaccination included need to be compliant with routine practice of Sri Lanka (23, 16.3%), free availability (11, 7.8%), fear of social isolation (11, 7.8%), and persuasion by the health authorities (7, 5%).

Majority of participants believed that children of both genders need to be vaccinated with equal priority (132, 93.6%). Nearly 70% (n = 101) believed that vaccinating a child is important for the health of the community while a similar number (n = 99, 70.25) also believed that vaccinating against uncommon diseases is also important.

Participants identified that midwives (135, 95.7%), doctors (97, 68.8%), books (110, 78.0%), TV (100, 70.9%), relatives (105, 74.5%) and online material (22, 16.6%) as modes of acquiring information about vaccination. Table 2 summarizes the key areas assessed and their responses to the statements.

**Table 2:** Parental knowledge on vaccination

Statement	Correct/yes	Incorrect/no	Do not know
General			
Vaccine provides nutritional supplement	51 (36.2%)	69 (48.9%)	21 (14.9%)
Vaccines are growth factors	90 (63.8%)	36 (25.5%)	15 (10.6%)
Vaccines are given to prevent infectious diseases	134 (95.0%)	5 (3.5%)	2 (1.4%)
Vaccines help the brain development of children	84 (59.6%)	36 (25.5%)	21 (14.9%)
Vaccine prevent non-communicable diseases	75 (53.2%)	46 (32.6%)	20 (14.2%)
Vaccinating your child indirectly prevent other children from getting the same disease	53 (36.9%)	76 (53.9%)	13 (9.2%)
Vaccines included in the Sri Lankan EPI include			
Oral polio vaccine (OPV)	134 (95.0%)	1 (0.7%)	6 (4.3%)
Pentavalent vaccine	59 (41.8%)	17 (12.1%)	65 (46.1%)
Measles, mumps and rubella (MMR) vaccine	120 (85.1%)	10 (7.1%)	11 (7.8%)
Japanese encephalitis (JE) vaccine	100 (70.9%)	9 (6.4%)	32 (22.7%)
Human papilloma virus (HPV) vaccine	39 (27.7%)	22 (15.6%)	80 (56.7%)
Influenza vaccine	68 (48.2%)	29 (20.6%)	44 (31.2%)
Varicella zoster vaccine	99 (70.2%)	22 (15.6%)	20 (14.2%)
Rabies vaccine	53 (37.6%)	74 (52.5%)	14 (9.9%)
Hepatitis A vaccine	64 (45.4%)	20 (14.2%)	57 (40.4%)
Matched first dose of the vaccine			
OPV—2 months	97 (68.8%)	30 (21.3%)	14 (9.9%)
Pentavalent vaccine—4 months	42 (29.8%)	8 (5.7%)	91 (64.5%)
MMR vaccine—12 months	53 (37.6%)	26 (18.4%)	62 (44.0%)
JE vaccine—12 months	44 (31.2%)	17 (12.1%)	80 (56.7%)
HPV vaccine—10 years	12 (8.5%)	11 (7.8%)	118 (83.7%)
Do you take your child for vaccination if he or she had the following conditions			
Cold	21 (14.9%)	116 (82.3%)	4 (2.8%)
Fever	9 (6.4%)	129 (91.5%)	3 (2.1%)
Diarrhoea	19 (13.5%)	101 (71.6%)	21 (14.9%)
If a vaccine dose is missed			
That vaccine should be avoided for life	17 (12.1%)	110 (78.0%)	14 (9.9%)
Vaccination schedule should be re-started from the beginning	21 (14.9%)	99 (70.2%)	21 (14.9%)
The required action will depend on the vaccine	88 (62.4%)	17 (12.1%)	36 (25.5%)
The required action will depend on the reason for the missed dose	82 (58.2%)	20 (14.2%)	39 (27.7%)
Attend the next available routine clinic and seek medical advise	135 (95.7%)	0	6 (4.3%)

A knowledge score was calculated considering 30 questions assessing the level of knowledge. Marks ranged from 5 to 24 with an average of 15 (SD 3.6). This made 62 (44.0%) of the participants to have above average, 13 (9.2%) to have average and 66 (46.8%) to have below average knowledge. More mothers had average (69, 57.5%) or above marks than fathers (6, 28.6%) and those who were educated up to A/L or above had a significantly higher proportion of above average marks (48, 64.9%) than those who were educated up to O/L or below (27, 40.3%) (Table 3). Employment status of the parent, having more children, income status and ethnicity had no significant association with the knowledge score.

**Table 3:** Factors associated with knowledge regarding immunization

Parameter	Average or above average score n, % or mean (SD)	Below average score n, % or mean (SD)	OR (95% CI)/P
Age	29.2 (5.0)	30.4 (5.9)	0.16
Sex			
Female	69 (57.5%)	51 (42.5%)	3.4 (1.2–9.3)
Male	6 (28.6%)	15 (71.4%)	
Ethnicity			
Sinhala	55 (52.9%)	49 (47.1%)	OR not calculated P for Fisher’s Exact test—0.314
Moor	18 (60.0%)	12 (40.0%)	
Tamil	2 (28.6%)	5 (71.4%)	
Level of education			
O/L or below	27 (40.3%)	40 (59.7%)	2.7 (1.4–5.4)
A/L or above	48 (64.0%)	26 (39.4%)	
Employment status of parent			
Employed	20 (48.8%)	21 (51.2%)	1.3 (0.6–2.7)
Unemployed	55 (55.0%)	45 (45.0%)	
No of children			
One	29 (50%)	29 (50%)	0.8 (0.4–1.6)
More than one	46 (55.4%)	37 (56.1%)	
Monthly income (Rs)^a^			
< 25,000	13 (43.3%)	17 (56.7%)	OR not calculated P for Fisher’s exact test—0.25
25,000–50,000	37 (52.1%)	34 (47.9%)	
> 50,000	23 (63.9%)	13 (36.1%)	

^a^Denominator 137

Two participants acknowledged that they had delayed at least one vaccine. No one stated that he/she plans to delay MMR vaccine while one stated possible delaying of the JE vaccine. One person stated that he/she had not given one vaccine to a child but declined to give the reason while one person stated that he/she delayed a vaccine dose due to an employment related issue.

Five (3.5%) participants stated that they were not in favour of vaccination despite bringing their children to get vaccinated. Four of them believed vaccines have more harmful effects than benefits, one stated that previous unpleasant experience of vaccination contributed to this view, two of them thought vaccination is not important for the health of children and one stated that their socio-cultural background does not favour vaccination. However, two of the five acknowledged that they did not have sufficient knowledge to make an informed decision about vaccination. Furthermore, 37 (26.2%) believed that it is better for their children to develop immunity by natural disease. Of the five participants who stated that they were not in favour of vaccination, two were males (fathers) and three were females (mothers), two of the five were Muslims while three were Sinhalese. One of these five was a graduate while two each had studied up to A/Ls and O/Ls. Income wise, two participants were from families with > 50,000 Rs monthly income, one from a family with an income between 25,000 and 50,000 Rs and two were from families with income < 25,000 Rs. Globally, vaccine hesitancy is known to be associated with difference factors such as affluence, religious and cultural beliefs [6, 7]. Our results demonstrate that those who expressed concerns were coming from diverse backgrounds. A qualitative study would shed more light into the exact factors that contribute to the development of vaccine hesitancy. These are likely to be different from those found in Western, developed countries.

Nine stated that they would not promote vaccination to others. However, a vast majority (131, 92.9%) believed that the EPI programme has been extremely useful to their children’s health.

Despite the small sample size, our findings indicate that knowledge regarding the childhood immunization programme of Sri Lanka and generally on vaccination among parents can be improved. Our findings highlight the need to involve both parents in such educational programmes and the need to target them at the level of understanding of the parents. The sources of information identified in this study indicates that medical professionals need to be more engaging in this despite their busy schedules at vaccination clinics.

The findings also highlight that even this sample of parents who had brought their children for vaccination have misconceptions about immunization. This number is likely to be commoner in the community level and is a reason for to be concerned. Lack of knowledge is one factor that contributes to vaccine hesitancy [7]. Further, this leaves parents vulnerable for mis-information given by anti-vaccine movements. Exposure to anti vaccine movement is shown to be associated with a reduced intention for vaccination [8]. Our findings further highlight that even in countries with high vaccine coverage, population is vulnerable to the potential influence of anti-vaccine movement, due to lack of proper understanding on vaccination and that timely, target specific educational programmes are a need of the hour.

## Limitations

This study was conducted using a small sample of 141 parents. The questionnaire used was developed for this study and the analysis method used was custom developed. However, similar approaches have been taken for other studies assessing knowledge scores. This needs to be extended to include higher number of parents representing both genders and all ethnicities in Sri Lanka. Furthermore, we did not assess in depth, the reasons for not being in favour of vaccination. A qualitative approach would be better suited for this. This study was conducted among parents who brought their children for vaccination, and should be extended to the community where the rates for vaccine hesitancy may be higher.
